# Eliciting Response Bias Within Forced Choice Tests to Detect Random Responders

**DOI:** 10.1038/s41598-019-45292-y

**Published:** 2019-06-19

**Authors:** Robin Orthey, Aldert Vrij, Ewout Meijer, Sharon Leal, Hartmut Blank

**Affiliations:** 1 Department of Psychology, University of Portsmouth, PO1 2DY Portsmouth, United Kingdom; 2 Faculty of Psychology & Neuroscience, Maastricht University, 622MD Maastricht, The Netherlands

**Keywords:** Diagnosis, Signs and symptoms

## Abstract

The Forced Choice Test (FCT) can be used to detect malingered loss of memory or sensory deficits. In this test, examinees are presented with two stimuli, one correct and one incorrect, in regards to a specific event or a perceptual discrimination task. The task is to select the correct answer alternative, or guess if it is unknown. Genuine impairment is associated with test scores that fall within chance performance. In contrast, malingered impairment is associated with purposeful avoidance of correct information, resulting in below chance performance. However, a substantial proportion of malingerers intentionally randomize their responses, and are missed by the test. Here we examine whether a ‘runs test’ and a ‘within test response ‘bias’ have diagnostic value to detect this intentional randomization. We instructed 73 examinees to malinger red/green blindness and subjected them to a FCT. For half of the examinees we manipulated the ambiguity between answer alternatives over the test trials in order to elicit a response bias. Compared to a sample of 10,000 cases of computer generated genuine performance, the runs test and response bias both detected malingered performance better than chance.

## Introduction

The Forced Choice Test (FCT) can be used to detect feigned memory loss for events^1–3^. In a FCT, an examinee is presented with a number of questions about the event, and each question is presented with two answer alternatives of which only one is correct. The examinee is instructed to select the correct answer to each question or to guess if they do not know the correct answer. The idea behind this test is that if an examinee truly has no recollection of the event, the total test score will fall within chance levels. Malingerers tend to purposefully select incorrect answer alternatives, and are more likely to obtain test scores lower than predicted by chance (so called underperformance). Similarly, FCTs can be used to detect sensory dysfunction, e.g., deafness^4^. Here, the examinee is presented with a series of trials, on half of which a sound is presented. When asked whether a sound was played, malingerers are more likely to underreport the number of correct answers. Laboratory studies investigating the detection accuracy for underperformance in FCTs show that the correct detection rate of malingerers - varies between 40% and 60%^5–11^, while the correct detection rate of genuine performers - is around 95%^5,7,9–11^.

The detection rates estimates outlined above corresponds to the prevalence of the specific strategies malingerers employ to avoid detection. Specifically, three hierarchical strategy levels predict different types of test scores^9,10^. Each level is based on the belief the examinee holds over the test’s detection mechanism. Based on this belief each strategy level is associated with a distinct response strategy. Specifically, Level 0 is associated with compliance with the test instructions, which results in endorsement of correct answers. This strategy occurs rarely (<5%)^9–11^. Level 1 strategies are based on the belief the test is designed to detect level 0 strategies, resulting in a counter-response such as selecting the incorrect answers instead. Approximately 40% of the participants report having used Level 1 strategies^9–11^. Level 2 strategies are based on the belief that the test is designed to detect level 1 strategies and predicts a counter-response, such as providing a mixture of correct and incorrect answers, so that test scores fall within chance performance. Level 2 strategies are most prevalent (around 45–50%)^9–11^. The traditional FCT criterion focuses on underperformance, i.e., choosing the incorrect answer more often than predicted by chance^3,12^. Hence, it is well suited for detecting level 1 strategies, but not suitable for detecting levels 0 and 2 strategies. This means that in order to increase the correct detection rate of malingerers it is important to improve the detection rates for level 2 strategy users, as they make up the majority of (undetected) malingerers.

Examinees employing a level 2 strategy attempt to simulate patterns of randomness. To detect this, the ‘runs test’ has been suggested. The criterion in this test is the number of alternations between correct and incorrect answers. It is based on the consistent finding that humans produce more alternations (≈60% alternation rate) than expected by chance (≈50% alternation rate) when trying to generate a random sequence of two options^13–15^. Previous studies have reported limited success using the runs test, identifying only a fraction of malingerers^6,16^. A likely reason for the poor diagnostic validity in these studies is a lack of power^10^. The alternation likelihood of real chance performance (50%) and alternations generated by humans (≈60%) are too similar to elicit statistically significant differences in short tests. This systematic difference becomes visible only in tests containing a sufficient number of items. In the current study, we implement the runs test on a considerably longer FCT than in previous studies, hypothesizing that the runs test becomes diagnostic with larger test sizes.

Aside from the runs criterion, we also explore the possibility of introducing an additional criterion specifically designed to detect level 2 strategies. This idea draws on the principle of *performance curves*, which describe the natural decline of performance over test items with increasing difficulty^17–20^. Frederick and Foster^20^ examined malingered cognitive deficits with a FCT of 100 trials in which the examinee had to identify relationships among abstract figures. The difficulty ranged from items so easy that even patients with genuine cognitive impairment could get the correct answer, to items so difficult that the likelihood of unimpaired examinees’ selecting the correct answer equalled chance performance. Even though the length and slope of this performance decline may differ between individuals, they all share the same pattern, namely that performance gradually declines with increasing difficulty. Interestingly, this was not the case for malingerers, who performed worse than chance on easy items and trended towards chance performance on items with increasing difficulty. Performance curves can also be introduced in a FCT by breaking it up into separate segments. Hiscock and Hiscock^21^ report the case of a patient whom they suspected of malingering. He was asked to memorize a five-digit number and to identify it among two alternatives after a short retention interval. The task was divided in three blocks of 24 trials with retention intervals of five seconds in the first block, ten seconds in the second block and 15 seconds in the last block. The task was designed to be so easy that the retention interval had no effect, evidenced by the performance of a five-year old, who showed above chance level performance for all three intervals. The patient displayed chance performance in the first block and below chance performance in the second and third block. Consequently, the authors suggest that malingerers adjust their test performance relative to the perceived difficulty of the test.

So far, the effect of performance curves has only been investigated for the underperformance criterion. Instead, we test whether a new criterion that produces a performance curve as a function of the perceived – but not the actual – difficulty, is sensitive to level 2 strategy users, i.e. those who randomize between correct and incorrect answers. Take, for example, a standard FCT to detect malingered red/green blindness. On each trial, an examinee is presented with a red and green square, and asked to select the green one. Malingerers using level 2 strategies, would select red and green squares approximately equally often, resulting in a total score within chance performance. If we vary the opacity - the transparency - of the red and green objects over trials, the examinee must not only take into consideration how many correct and incorrect answers were selected, but also at what opacity. Hence, malingerers could differ from chance performance by displaying a preference to avoid/endorse correct answers relative to the perceived difficulty of the trials. Perceived difficulty was used, because it can be introduced as an orthogonal factor to the malingered cognitive deficits. So, the task may look more/less difficult, but would have no effect on genuinely impaired performance. Malingerers are expected to be unable to have an accurate estimate of how an actually impaired examinee would respond, an effect other malingering tools such as the Structured Inventory of Malingered Symptomatology^22–24^ make use of as well. Consequently, an examinee may, for example, think that on trials with strong opacity, the difference between the two objects is so clear that even red/green blind participants will perceive the difference, and select the correct alternative. This would result in a correlation between correct/incorrect answers and opacity, and this correlational response bias can serve as a new criterion specifically designed to detect intentional randomization.

In the current experiment, we asked examinees to malinger red green blindness and subjected them to one of two conditions: a standard FCT or a FCT where perceived difficulty varied per trial. Perceived difficulty was induced by varying the opacity of the stimuli over trials. We chose malingered red/green blindness for two reasons. First, perceived difficulty could be manipulated easily and objectively through opacity. Second, red/green blindness is by definition associated with chance performance, not just a steep decline in ability. Therefore, response for genuine red/green blindness could be generated through computer simulation. We evaluated three measures to detect examinees employing level 2 strategies, i.e., who employ intentional randomization of correct and incorrect answers. We only analyse examinees using level 2 strategies, and therefore expect that the number of correct alternatives selected will fail to distinguish malingered from genuine red/green blindness (Hypothesis 1). Our FCT consists of 100 trials, which is the same test length often used to assess the human ability to generate randomness^13–15^, and considerably larger than what has been employed in previous studies^6,16^. For that reason, we expect the runs test - based on the number of alternations between correct and incorrect - to detect malingerers using a level 2 strategy better than chance, with higher alternation rates indicating malingered performance (Hypothesis 2). Additionally, we expect biased responding as a function of the varying degree of opacity. We refer to this bias simply as *response bias*, and expect this to detect malingerers better than chance (Hypothesis 3).

## Method

### Participants

We tested 84 examinees from a university undergraduate population. Genuine red/green blindness was an exclusion criterion and zero examinees were excluded for this reason. Five examinees were excluded, because they disregarded the instructions, leaving 79 remaining. As this experiment examines examinees who choose a level 2 strategy of intentionally randomizing correct and incorrect answers, we excluded all participants who reported using a different strategy. As a consequence, we excluded six (four in the Standard condition and two in the Opacity condition), leaving 37 in the Standard condition and 36 in the Opacity condition. Of these 73 examinees, 53 were female, 20 were male. Their *mean* age was 23.00 (*SD* = 6.61) and all examinees were at least 18 years old. Examinees were rewarded for their participation with 5 euros or course credit. Ethical approval was obtained from the Ethics Review Committee Psychology and Neuroscience (ERCPN) from Maastricht University. The experiment was performed in accordance with relevant guidelines and regulations and informed consent was obtained.

### Procedure

All examinees were instructed to feign red/green blindness. To do so we provided them with some information about red/green blindness. In essence, examinees received information that both red and green look like grey to someone with genuine red/green blindness. The information was made to look like it was derived from Wikipedia^25^. In addition, examinees were told that a number of tests would follow to establish whether their alleged red/green blindness was genuine. The warning was issued in order to facilitate the adoption of level 2 strategies as seen in previous studies^10,16^. Its effectiveness was reflected in the small number of examinees reporting level 0 or level 1 strategies.

The test started with two filler tasks such that the FCT was embedded into a credible task battery. First, we asked examinees to give a brief written statement indicating how red/green blindness has negatively affected them in their life. After that we administered three Ishihara plots that consist of a number of differently coloured circles. The hues are chosen in a way that colour blind and examinees without visual impairment see different numbers. Each plot was provided with two answer alternatives. One was the number people with red/green blindness would have seen and the other was the number unimpaired people would have seen^26^. No data were recorded on both tasks.

Then, examinees were subjected to the FCT examination on a computer and randomly assigned to either the Standard or Opacity condition. In the Standard condition, examinees were informed that in the next part they would be presented with red and green squares and were instructed to always indicate the green square. Each trial had the same structure. First, in the middle of the screen the instruction to select the green square was presented and at the bottom centre was a ‘next’ button located. Once examinees clicked the next button the instructions disappeared and two equal sized rectangles appeared at the top of the screen. The rectangles were in their entirety red (RGB = 255,0,0) or green (RGB = 0,255,0). Examinees could indicate their choice by clicking within the particular rectangle with a mouse. The horizontal location (left/right) of the green square was determined randomly on each trial. In total 100 of these trials were presented to each examinee.

In the Opacity condition we manipulated the opacity of both rectangles. Opacity refers to how see-through the rectangles were and can range from 100% - not see-through at all - to 0% - completely vanished -. In essence, with lower opacity it becomes harder to perceive the colour of both rectangles. Out of the 100 trials, 10 featured 100% opacity and were identical to the trials in the control condition. The remaining 90 trials featured opacities from 10% to 99% in increments of 1%. We chose to omit trials with opacities lower than 10%, to make sure people with normal vision can still reasonably be expected to perceive the colour of the stimuli. The order of presentation was randomized over all 100 trials.

After the FCT, examinees were told that the assessment was over and that they should answer everything honestly. Then examinees were asked the following question: “What did you do during the test procedure to make the investigator believe that you are actually red-green color blind?” Their response was recorded, transcribed and coded by two independent coders (see below).

Finally we presented all examinees with 20 trails featuring 100% opacity. Their task was to honestly indicate the green rectangle on each trial. This served as a performance check. Examinees who made one or more mistakes on this task were excluded. Zero participants were excluded for this reason.

### Design

Three dependent variables were used. We computed the correct scores by summing the number of trials where the correct answer alternative was selected. For the ‘runs test’ we computed the number of alternations between correct/incorrect items. Both scores were transformed into z-scores according to the binomial distribution^27^. Hence, the z-scores indicated how (un)likely the raw score was to occur through chance. For the runs test, these z-scores were then multiplied by −1 so that for all criteria lower scores were indicative of malingered performance. In the Opacity condition, we estimated the response bias by conducting within each examinee a t-test for a point-biserial correlation with their choice (correct/incorrect) on each trial and the corresponding opacity used in that trial. As a result, we obtained for each examinee a correlation, indicating the strength and direction of the bias, and a p-value, indicating the significance of the correlation. We used the p-value as criterion for the response bias as the smaller the p-value was, the more unlikely the response bias was to occur through chance. We chose the p-value over the correlation because, unlike correlations, p-values can only be positive.

Examinees who are truly colour blind can be expected to show random performance. We therefore compared the distribution of our malingered examinees to a simulated random distribution. Thus, this experiment featured a 2 red/green blindness (Malingered vs Genuine) × 2 Opacity (Standard vs Opacity) between-subjects design. We simulated the response patterns of the genuine red/green blindness group for the Standard and Opacity condition. Each response pattern was simulated at the trial level. By using random numbers we determined on each trial whether a participant would select a correct or incorrect response with a 50% probability each. Random numbers were generated using atmospheric noise^28^. With these random numbers we simulated choices as if an examinee was guessing on each trial. We computed the three dependent variables the same way as for the malingerers. In total, 5000 responses were generated for genuine red/green blindness in the Standard and Opacity condition each.

We report the validity of the three dependent variables in terms of Signal Detection Theory parameters^29^, specifically using the Area Under the Curve (AUC) of the Receiver Operating Characteristic (ROC). The ROC plots the correct detection rate for malingered performance against 1 – the correct detection rate for genuine performance for all possible cut-off points. The AUC represents the detection accuracy across all possible points, serving as a general measure of detection accuracy (for a more extensive explanation of Signal Detection Theory and the AUC see Hanley and McNeil, 1982^30^). The AUC ranges from 0 to 1, with 0.5 indicating chance performance. Values significantly higher than 0.5 suggest that the criterion has diagnostic value.

Examinees’ answers about their behaviour during the FCT were transcribed and coded into three strategy levels as previously^9,10^ suggested. These strategy levels were referenced to the original test instruction (‘Select the correct answer alternatives. If you don’t know, guess.’) and were defined as follows: A *Level 0* strategy forms no beliefs over the test’s classification mechanism and leads to compliance with the test instructions (i.e. overperformance). A *Level 1* strategy forms a belief based on the instructions and behaviour manifests as a reaction to it. The most common behaviour is intentional avoidance of correct information leading to underperformance. A *Level* 2 strategy is based on the belief that the test uses a *Level 1* classification mechanism and therefore test behaviour manifests as a reaction to a *Level 1* strategy. The most common behaviour is to attempt to provide a random mixture of correct and incorrect information.

## Results

**Figure 1:**
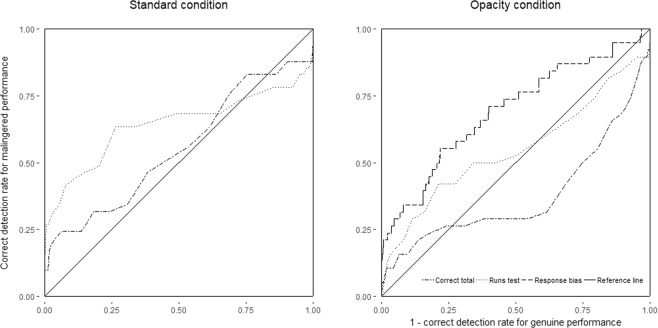
ROC curves for the Standard condition (left panel), and the Opacity condition (right panel).

Table 1 displays the detection accuracies using the correct scores, the runs test and the response bias as detection criteria, respectively. The ROC plots are given in Fig. 1. As hypothesized, the correct scores did not distinguish malingered from genuine red/green blindness in the Standard condition, *AUC* = 0.53, *p* = 0.527, *95% CI* [0.42 0.63]. In contrast, malingerers in the Opacity condition were detected with below chance level performance, *AUC* = 0.39, *p* = 0.033, *95% CI* [0.28 0.51]. This supports our first hypothesis that the underperformance criterion has no predictive validity for examinees randomizing between correct and incorrect answers.

**Table 1:** Detection accuracies for all criteria for the Standard and Opacity condition.

Condition	Criterion	AUC	*p*	95% CI
Standard	Correct total	0.53	0.527	[0.42 0.63]
	Runs test	0.69	<0.001	[0.57 0.81]
Opacity	Correct total	0.39	0.033	[0.28 0.51]
	Runs test	0.58	0.101	[0.47 0.69]
	Response Bias	0.70	<0.001	[0.61 0.79]

**Figure 2:**
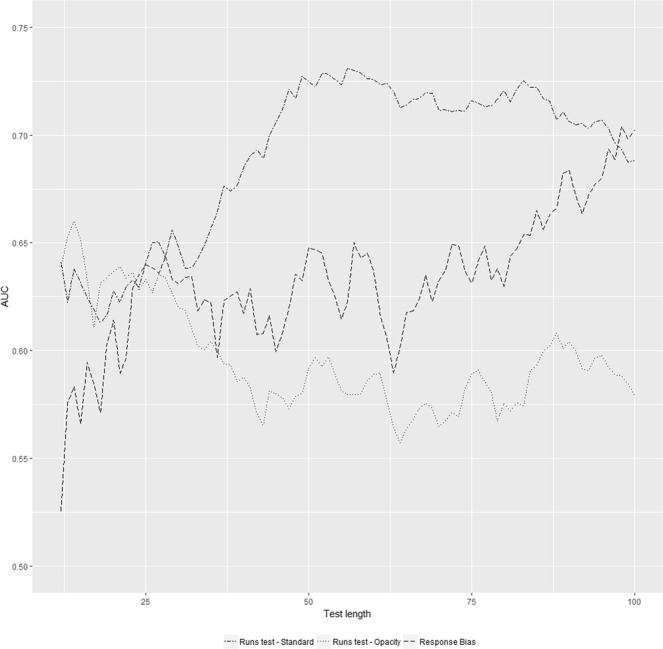
Detection accuracy over the length. AUCs of the Runs test in the Standard and Opacity condition as well as the Response bias in the Opacity condition for all test lengths from 12 to 100 trials.

The runs test detected malingerers in the Standard condition, *AUC* = 0.69, *p* < 0.001, *95% CI* [0.57 0.81], but not in the Opacity condition, *AUC* = 0.58, *p* = 0.101, *95% CI* [0.47 0.69], better than chance. Hence, there was only partial support for our second hypothesis that the runs test can detect examinees randomizing between correct and incorrect answers. To further estimate the relationship between test length and detection accuracy we computed the AUC for all test lengths by taking the first n trials, with n varying from 12 to 100 (see Fig. 2). The trend suggested that in the Standard condition detection accuracy increases with test size and peaked at a test size between 50 to 70 trials. In the Opacity condition the detection accuracy of the runs test declined with test length continuously.

Finally, we assessed the validity of the response bias in the Opacity condition. We used the p-value as a continuous criterion as it indicates how (un)likely a response pattern is to occur through chance. The AUC was estimated using lower scores as indicative of malingering. We found that this criterion differentiated malingered from genuine red/green blindness better than chance, *AUC* = 0.70, *p* < 0.001, *95% CI* [0.61 0.79]. We also checked how many of our participants showed a significant response bias. This was the case for 27.8% (p < 0.05). Of this 27.8%, 60% displayed a positive correlation (*mean* = 0.45, *SD* = 0.17) and 40% displayed a negative correlation (*mean* = −0.32, *SD* = 0.22). As expected, of the simulated genuine red/green blindness 5% was erroneously classified as malingerer. A chi-square test indicated that the 27.8% correct detection rate of malingerers differed significantly from the 5% expected by chance, *Χ*^*2*^ = (1, N = 5036) = 38.49, *p* < 0.001. Furthermore, when calculated over all possible test lengths (see Fig. 2), the AUC of the response bias increased gradually with test length and peaked at 100 trials. These findings support our third hypothesis that the response bias can serve as a valid indicator of malingering.

## Discussion

This study examined the diagnostic value of correct total scores, the runs test and the response bias criteria to detect malingered red/green blindness in examinees who utilize level 2 strategies, i.e., who randomize between correct and incorrect answers, in a Forced Choice Test. In the Standard condition all trials were identical, but in our Opacity condition we varied the opacity of both stimuli over all trials in order to tempt malingerers into adjusting their alternations between correct and incorrect answers according to the opacity of the trials. The purpose of this manipulation was to elicit an additional response bias that could serve as a new criterion to detect those who employ level 2 strategies.

The results in the Standard condition suggest that the runs test has diagnostic value, provided the test size is large enough. This finding is encouraging for those applications of the FCT where the number of trials that are included in the test is unbound, such as cases of cognitive deficits. For alleged memory, auditory, or visual impairments, trials can be easily generated and repeated. It has less relevance, however, in situations where the trials are specific to unique pieces of information, for example in cases of autobiographical memory loss^6,16^. Moreover, the figure plotting the validity for the different test lengths indicates a potential test length around 50 to 70 trials, after which the accuracy of the runs test decreases. Future studies could help investigate whether this finding replicates, and help pinpoint the optimal test length for this criterion.

The effectiveness of the ‘runs test’ was limited to the Standard condition, and not present in the Opacity condition. Instead, the response bias proved a valid indicator of malingering. As seen in Fig. 1, the detection accuracy of the response bias gradually increased, while the detection accuracy of the runs test gradually decreased. A potential reason for the ineffectiveness of the runs test could be that the response bias, in form of the varying opacities, is very salient and malingerers preferred to calibrate their response pattern in regards to opacity, rather than with regard to their alternation rate between correct and incorrect answers. This finding is relevant because it suggests that response biases can be elicited through perceived difficulty. This may make performance curve decision models much more resistant to countermeasures, as the malingerer must firstly determine whether the subsequent trials just appear more/less difficult or actually are more/less difficult for genuine impairment. Consequently, the runs test and response bias are suitable follow-up criteria for the underperformance criterion^10^. In this procedure the test score would be evaluated for underperformance, and if undetected subjected to a criterion sensitive to intentional random responding. The benefit of this procedure is that the FCT becomes sensitive to the vast majority of malingerers as opposed to subgroups representative of 40–50% of malingerers when using only a single criterion. Future research may also attempt to combine both types of response bias for even better detection accuracy.

Implementing a response bias to detect malingering features two challenges: (i) The introduced bias must be varied and measured objectively. In cases of alleged malingered sensory deficits such as visual or audio impairment, degrading/enhancing the stimuli can easily be done objectively. In case of malingered memory loss, the perceived importance of questions could be manipulated, but this would be challenging to do objectively. (ii) The test must contain a sufficient number of trials for the statistical assessment of the response bias. This can easily be done for malingered sensory deficits as trials can be repeated as often as necessary. This becomes a problem for cases of malingered memory loss as events often contain only few pieces of information^31^ and malingerers must also remember the information. Thus, in terms of practical application, the response bias criterion seems best suited for malingered sensory deficits and less so for cases of malingered memory loss.

Using simulated data to represent genuine performance may raise the concern that this limits the ecological validity of our findings. Previous simulation of control group behaviour^32^ has been shown to be a poor reflection of real clinical samples^33^ in estimating incorrectly classified genuine performers as a function of increasing the number of tests used to detect malingered performance. Larrabee^34^ argues that the performance of real clinical samples resembles a ceiling effect (the majority of the sample displays almost perfect performance), rather than a standard normal distribution with a mean of 0 and standard deviation of 1 as used for the simulation. We recognize these concerns, but argue that they do not apply in this case for two reasons: (i) In a FCT, by definition, stimulus pairs featured in the trials are indistinguishable for examinees with genuine impairment. Therefore, genuine performance follows the chance distribution for all three criteria, which means the test behaviour and not only can the test result be simulated. From this follows that characteristics of the sample can be expected to be representative of reality. (ii) Furthermore, a meta-analysis of the Concealed Information Test, a test that also relies on a known distribution, suggests that simulating data is even better, as it reduces sampling biases caused by small group sizes^35^.

Another concern may be that the increase in detection accuracy is related to statistic fundamentals. With increased sample size the p-values of the t-tests for point bi-serial correlation become smaller automatically. While this is true, it is important to realize that this only applies to the malingerer. Genuine guessing can be expected to always produce the same equal distribution of p-values, regardless of test length. In contrast, with increasing test length weaker effects within the malingerer population yield smaller p-values. As a consequence, detection accuracy of the criterion increases, that is at least until all malingerers that do exhibit a response bias are detected. Therefore, the effect of test length on the response bias in examinees using level 2 strategies is not trivial.

In sum, our findings suggest that examinees employing level 2 strategies in a FCT can be detected by the runs test, provided the FCT features enough trials, or by varying perceived difficulty and testing for a systematic response bias. As level 2 strategies typically remain undetected and are the most common type of strategies, these new criteria can be used to increase the overall detection accuracy of FCTs.

## Data Availability

The dataset generated and analysed during the current study is accessible on the Open Science Framework (see https://osf.io/4v2fd/?view_only=5c1a58ce79024576a4995267debf4aae).
